# Nighttime light exposure is associated with metabolic dysfunction in schizophrenia: a cross-sectional analysis of the LENS study

**DOI:** 10.1093/sleep/zsag177

**Published:** 2026-06-29

**Authors:** Rina Taniguchi, Yuichi Esaki, Kenji Obayashi, Keigo Saeki, Soji Tsuboi, Kiyoshi Fujita, Nakao Iwata, Tsuyoshi Kitajima

**Affiliations:** Department of Psychiatry, Okehazama Hospital, Toyoake, Aichi, Japan; Department of Psychiatry, Okehazama Hospital, Toyoake, Aichi, Japan; Department of Psychiatry, Fujita Health University School of Medicine, Toyoake, Aichi, Japan; Department of Epidemiology, Nara Medical University School of Medicine, Nara, Japan; Department of Epidemiology, Nara Medical University School of Medicine, Nara, Japan; Department of Psychiatry, Okehazama Hospital, Toyoake, Aichi, Japan; Department of Psychiatry, Okehazama Hospital, Toyoake, Aichi, Japan; The Neuroscience Research Center, Toyoake, Aichi, Japan; Department of Psychiatry, Fujita Health University School of Medicine, Toyoake, Aichi, Japan; Department of Psychiatry, Fujita Health University School of Medicine, Toyoake, Aichi, Japan

**Keywords:** schizophrenia, metabolic syndrome, light, actigraphy, obesity

## Abstract

**Study Objectives:**

Accumulating evidences have investigated the impact of nighttime light exposure on metabolic dysfunction, including obesity, hypertension, diabetes, and dyslipidemia, in the general population. However, no study has investigated this effect in patients with schizophrenia. Therefore, this study aimed to investigate the association between nighttime light exposure and metabolic dysfunction in patients with schizophrenia.

**Materials and Methods:**

This was a cross-sectional study including 222 outpatients with schizophrenia enrolled in the Light Exposure and Neurobiology in Schizophrenia study. Nighttime light exposure was objectively evaluated using a wrist-worn actigraph and a bedside photometer over seven consecutive days. Physical examinations and blood sampling were performed at the clinic.

**Results:**

After adjusting for potential confounding factors, multivariable logistic regression analysis revealed that the participants with the highest quartile of nighttime illuminance measured using actigraphy had significantly higher odds ratios (OR) for obesity (OR, 4.01; 95% confidence interval [CI], 1.69–9.53), hypertension (OR, 3.38; 95% CI, 1.40–8.18), and dyslipidemia (OR, 2.41; 95% CI, 1.01–5.74) than those with the lowest quartile. No significant association was observed between nighttime illuminance and OR for diabetes. However, increased nighttime illuminance was significantly associated with higher glycosylated hemoglobin levels. A significant association was observed between nighttime illuminance measured using the bedside photometer and OR for hypertension.

**Conclusions:**

We found a significant association between nighttime light exposure and metabolic dysfunction in patients with schizophrenia. Further longitudinal studies are needed to clarify this association.

**Statement of Significance**

According to recent large cohort studies involving the general population, nighttime light exposure is associated with metabolic dysfunction, including obesity, hypertension, diabetes, and dyslipidemia; however, this effect remains uninvestigated in patients with schizophrenia. Therefore, this study aimed to investigate the association between nighttime light exposure and metabolic dysfunction in patients with schizophrenia. Results showed that increased nighttime light exposure was significantly associated with higher odds ratios for obesity, hypertension, and dyslipidemia in such patients. Additionally, increased nighttime illuminance was significantly associated with higher glycosylated hemoglobin levels. Further longitudinal research is warranted to explore the nature of this observation.

## Graphical Abstract

**Graphical Abstract:**
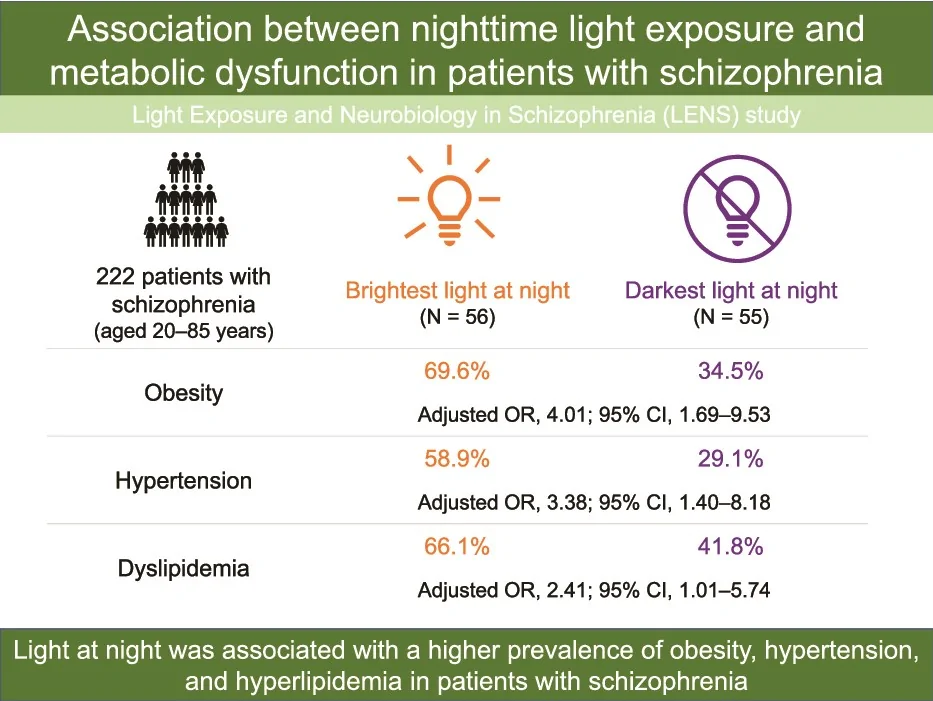

## Introduction

Schizophrenia is a severe, chronic neuropsychiatric disorder characterized by positive symptoms, such as delusions and hallucinations, and negative symptoms, such as blunted affect and social withdrawal [1]. Metabolic dysfunction, including obesity, hypertension, diabetes, and dyslipidemia, is common among individuals with schizophrenia, affecting approximately one-third of patients with schizophrenia globally, which is approximately twice the prevalence in the general population [2, 3]. Metabolic dysfunction is associated not only with cardiovascular disease and mortality but also with increased risk of psychotic relapse and cognitive impairment in patients with schizophrenia [4–6]. Therefore, identifying factors associated with metabolic dysfunction in patients with schizophrenia is important.

The advent of artificial lighting technology has brought significant benefits to national economic and social development. However, it has profoundly changed the natural light/dark cycle [7]. Excessive nighttime light exposure has been reported to be associated with various adverse health problems, such as cardiovascular diseases, mental and sleep problems, cancer, and mortality risk [8–12]. Additionally, recent large cohort studies and meta-analyses of the general population and patients with bipolar disorder have shown that nighttime light exposure is associated with obesity, hypertension, diabetes, and dyslipidemia [13–21]. Therefore, nighttime light exposure may be associated with metabolic dysfunction in patients with schizophrenia.

Therefore, this study aimed to investigate the associations between nighttime light exposure and metabolic dysfunction, including obesity, hypertension, diabetes, and dyslipidemia, in patients with schizophrenia. We hypothesized that increased nighttime light exposure would be associated with a higher prevalence of metabolic dysfunction in patients with schizophrenia.

## Materials and methods

### Participants and procedure

This cross-sectional study included patients with schizophrenia who participated in the Light Exposure and Neurobiology in Schizophrenia (LENS) study. The LENS study is a prospective, naturalistic, observational study conducted in Japan from March 2024 to investigate the effect of light exposure on outpatients with schizophrenia. In this study, we extracted individuals aged ≥20 years and diagnosed with schizophrenia according to the Diagnostic and Statistical Manual of Mental Disorders (fifth edition). The exclusion criteria included night shift workers and those with serious symptoms as judged by a clinician. Participants underwent physical examinations and blood sampling at the clinic. They were then asked to wear an actigraph (Actiwatch Spectrum Plus; Respironics Inc., PA, USA) on the wrist of their nondominant arm 24 h a day, record bedtime and rising time in a sleep diary, and set a photometer (LX-28SD; Sato Shoji Inc., Kanagawa, Japan) at their bedside, near their head, at eye level for seven consecutive days. Participants with actigraphy measurement durations of <2 days were excluded from this study. Finally, 222 patients were included in the analysis. This study was approved by the Ethics Committee of Okehazama Hospital (approval number R05-027). Written informed consent was obtained from all participants. This study was registered with UMIN-CTR (identifier: UMIN000053763).

### Nighttime light exposure assessment

Nighttime light exposure was recorded using the actigraph and photometer at 1-min intervals throughout the period from bedtime to rising time, as recorded in their sleep diaries. The detection algorithm in the actigraphy software (Actiware, version 6.0.9, Respironics) automatically excluded any periods during which the actigraph was not worn. Nighttime illuminance was defined as the average illuminance measured using the actigraph and photometer between bedtime and rising time, as recorded in the sleep diary.

### Measurement of metabolic dysfunction

Physical examinations and blood samples were primarily performed in the morning. Participants’ height and weight were measured while wearing light clothing without shoes. Body mass index (BMI) was calculated as weight (kg) divided by height (m^2^). Abdominal circumference was measured at the level of the umbilicus in the standing position. Systolic blood pressure (SBP) and diastolic blood pressure (DBP) were measured using an automatic sphygmomanometer (Omron HEM-1040, Omron Healthcare, Inc., Kyoto, Japan) with the patients in the sitting position after at least 5 min of rest. A blood draw was performed for routine blood testing to measure plasma glucose, glycosylated hemoglobin (HbA1c), triglycerides (TG), low-density lipoprotein cholesterol (LDL-C), and high-density lipoprotein cholesterol (HDL-C). Participants’ adherence to fasting requirements for blood testing could not be confirmed. Therefore, plasma glucose and TG were treated as casual plasma glucose and non-fasting TG, respectively. Metabolic dysfunction was defined according to the latest Japanese guidelines. Obesity was defined as BMI ≥ 25 kg/m^2^ [22]. Hypertension was defined as SBP ≥ 140 mmHg, DBP ≥ 90 mmHg, or use of antihypertensive medication [23]. Diabetes was defined as a casual plasma glucose ≥200 mg/dL, HbA1c ≥ 6.5 percent, or use of antihyperglycemic medication [24]. Dyslipidemia was defined as TG ≥ 175 mg/dL, HDL-C < 40 mg/dL, LDL-C ≥ 140 mg/dL, or use of lipid-lowering medication [25].

### Covariates

Psychiatric symptoms were assessed using the Positive and Negative Syndrome Scale [26]. Sleep and daytime physical activity were objectively evaluated using the actigraph. Total sleep time was defined as the total time spent asleep from bedtime to rising time, excluding the time spent awake during the night. Daytime physical activity was defined as the average activity count/min between the rising time and bedtime. For each participant’s first day of measurement, the day length from sunrise to sunset in Aichi, Japan (latitude, 35°N) was obtained from the website of the National Astronomical Observatory of Japan. Data on the participants’ medications were collected from their clinical records. Antipsychotic doses were determined based on the risperidone-equivalent dose [27–29].

### Statistical analyses

Statistical analyses were performed using SPSS version 29.0 for Windows (IBM, Armonk, NY, USA) and R version 4.4.1 (R Project for Statistical Computing, Vienna, Austria). Two-sided *p*-values <.05 indicated statistical significance. Continuous variables were presented as medians and interquartile ranges (IQR), whereas categorical variables were presented as numbers and percentages. The mean values of seven consecutive days were used to analyze light exposure, sleep, and physical activity parameters. Participants were split into four groups based on the quartiles of nighttime illuminance. The association trend for the variables was evaluated using logistic regression or the Jonckheere–Terpstra test, as appropriate. The correlations between actigraphy and photometer measure for 7-day nighttime illuminance were evaluated using Spearman’s rank correlation coefficient (*rs*).

Logistic regression and analysis of covariance were performed to estimate the association between nighttime illuminance and metabolic dysfunction. In the multivariable models, the odds ratios (ORs) for obesity, hypertension, diabetes, and dyslipidemia were simultaneously adjusted for confounding factors associated with metabolic dysfunction, including age (≥60 or < 60 year), gender (male or female), current smoking status (yes or no), total sleep time (high or low, category based on the median value), daytime physical activity (per count/min), rising time (per hours), antipsychotic dose (per mg/day), and antidepressant use (yes or no) [30–35]. The associations between nighttime illuminance and metabolic parameters, including body weight, abdominal circumference, BMI, SBP, DBP, TG, HDL-C, and LDL-C, were investigated using analysis of covariance. For multiple comparisons, the Benjamini–Hochberg method in R was used for false discovery rate (FDR) correction.

To address the possibility of reverse causation and residual confounding, additional multivariable analyses were conducted adjusting for factors associated with nighttime light exposure, including age (per year), gender (male or female), current smoking status (yes or no), duration of illness (per year), total sleep time (per min), physical activity (per count/min), and antipsychotic dose (per mg/day).

## Results

**Figure 1:**
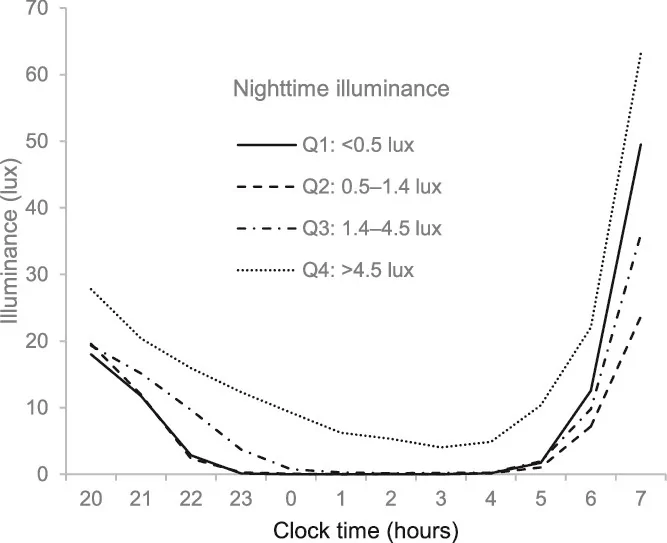
*Median illuminance from 20:00 to 7:00 for 222 patients with schizophrenia*. Solid, dashed, dashed-dotted, and dotted lines indicate participants exposed to a median illuminance of <0.5, 0.5–1.4, 1.4–4.5, and ≥ 4.5 lux (Q1, Q2, Q3, and Q4), respectively. Illuminance measured using an actigraph is plotted for each hour.

The median age of the 222 participants was 50 years (IQR 39–59 years). Of the 222 participants, 122 (55.0 percent) were males (Table 1). The median nighttime illuminance measured using the actigraph and photometer was 1.4 lux (0.5–4.5 lux) and 4.0 lux (1.0–15.4 lux), respectively (Table 1). As nighttime illuminance quartiles increased, age, proportion of current smokers, illness duration, and antipsychotic dose significantly increased, rising time significantly delayed, and daytime physical activity significantly decreased (Table 1). The prevalence of obesity, hypertension, diabetes, and dyslipidemia were 55 percent, 43 percent, 13 percent, and 49 percent, respectively (Table 2). Increased nighttime illuminance was significantly associated with the proportions of participants with obesity, hypertension, and dyslipidemia (Table 2). Consistently, as nighttime illuminance quartiles increased, body weight, abdominal circumference, BMI, SBP, DBP, TG, and LDL-C significantly increased, whereas HDL-C significantly decreased (Table 2). Additionally, nighttime illuminance was significantly associated with casual plasma glucose and HbA1c levels (Table 2). Figure 1 shows the median illuminance for each hour from 20:00 to 7:00. Participants in the highest nighttime illuminance quartile (Q4) were exposed to more light throughout the night than those in the lowest quartile (Q1). Moderate to high correlations were observed between actigraphy- and photometer-measured for 7-day nighttime illuminance (rs range: 0.55–0.70) (Table S1). Additionally, Table S2 presents the concordance matrix of Q1–Q4 classifications between the actigraphy- and photometer- measured nighttime illuminance categories. For actigraphy, the mean (standard deviation [SD]) and median (IQR) values of valid measurement nights were 6.66 (1.03) and 7 [7], respectively. The mean and median non-wear durations from bedtime to rising time were 3.9 (15.5) and 0 (0–0) min, respectively.

**Table 1:** Demographic and clinical characteristics and medications stratified by nighttime illuminance measured using an actigraph

		**Quartile groups of nighttime illuminance measured using an actigraph**				
**Characteristics**	**Total (n = 222)**	**Q1 n = 55 (<0.5 lux)**	**Q2 n = 56 (0.5 to 1.4 lux)**	**Q3 n = 55 (1.4 to 4.5 lux)**	**Q4 n = 56 (≥ 4.5lux)**	***P trend***
**Demographic characteristics**						
Age, years, median (IQR)	50.0 (39.0-59.0)	47.0 (33.0-54.0)	49.5 (39.0-60.8)	50.0 (44.0-59.0)	53.0 (41.0-61.0)	0.02
Gender, male, n (%)	122 (55.0%)	31 (56.4%)	32 (57.1%)	26 (47.3%)	33 (58.9%)	0.95
Married, n (%)	46 (20.7%)	11 (20.0%)	13 (23.2%)	14 (25.5%)	8 (14.3%)	0.53
Education (≥16 years), n (%)	52 (23.4%)	13 (23.6%)	13 (23.2%)	14 (25.5%)	12 (21.4%)	0.86
Employed, n (%)	76 (34.2%)	23 (41.8%)	21 (37.5%)	18 (32.7%)	14 (25.0%)	0.05
Current smoker, n (%)	47 (21.2%)	3 (5.5%)	17 (30.4%)	11 (20.0%)	16 (28.6%)	0.02
**Clinical characteristics, median (IQR)**						
Age at onset of schizophrenia, years	24.0 (20.0-33.5)	24.0 (19.0-32.0)	23.5 (20.0-32.0)	29.0 (21.0-37.0)	24.0 (19.0-32.0)	0.53
Duration of illness, years	20.0 (12.0-30.0)	18.0 (8.0-27.0)	18.5 (12.0-28.8)	22.0 (10.0-29.0)	23.0 (17.0-34.0)	0.01
PANSS, positive score	16.0 (14.0-18.0)	16.0 (14.0-18.0)	16.0 (14.0-18.0)	16.0 (14.0-19.5)	16.0 (14.0-18.0)	0.55
PANSS, negative score	19.0 (17.0-22.0)	19.0 (16.8-22.3)	18.5 (16.3-21.0)	19.0 (17.0-21.0)	19.0 (16.0-22.0)	0.67
PANSS, general score	38.0 (34.0-41.0)	38.0 (33.8-42.0)	37.5 (34.0-41.0)	38.0 (34.5-42.0)	37.0 (35.0-41.3)	0.61
PANSS, total score	74.0 (66.0-80.0)	74.5 (64.0-81.3)	72.0 (65.0-79.8)	75.0 (67.5-80.5)	74.0 (67.8-80.0)	0.66
Bedtime, clock time	22:05 (21:07-23:07)	22:02 (21:01-22:47)	21:33 (20:57-22:56)	22:16 (21:22-23:27)	22:44 (21:08-23:25)	0.05
Rising time, clock time	6:36 (5:39-7:41)	6:09 (5:19-7:05)	6:38 (5:41-7:25)	7:06 (5:53-8:10)	6:46 (5:40-8:15)	<0.01
Total sleep time, min, median (IQR)	426.9 (345.6-490.6)	425.1 (361.4-492.0)	439.1 (365.9-506.4)	427.9 (334.3-490.4)	396.0 (311.7-464.8)	0.05
Daytime physical activity, counts/min	146.9 (93.9-200.2)	158.2 (99.5-231.2)	151.1 (87.8-211.3)	148.2 (124.8-189.4)	119.2 (81.7-190.8)	0.04
Nighttime illuminance (actigraph), lux	1.4 (0.5-4.5)	0.3 (0.1-0.4)	0.8 (0.6-1.1)	2.7 (2.0-3.2)	9.1 (6.5-20.2)	<0.01
Nighttime illuminance (photometer), lux	4.0 (1.0-15.4)	0.7 (0.3-1.8)	2.4 (1.3-4.6)	7.6 (3.6-18.5)	20.4 (13.2-47.6)	<0.01
Day length, min	805.0 (734.5-855.0)	770.0 (675.0-843.0)	835.0 (766.5-868.8)	800.0 (736.0-855.0)	815.0 (735.8-852.8)	0.23
**Medication**						
Antipsychotics, n (%)	220 (99.1%)	55 (100.0%)	55 (98.2%)	54 (98.2%)	56 (100.0%)	0.99
Antipsychotics, mg/day, median (IQR)	6.0 (3.0-9.5)	4.5 (2.3-8.0)	6.9 (3.0-10.2)	5.0 (2.8-9.1)	8.0 (5.1-11.8)	<0.01
Antidepressant, n (%)	25 (11.3%)	6 (10.9%)	7 (12.5%)	10 (18.2%)	2 (3.6%)	0.38

Data are expressed as the median (interquartile range) or number (%). PANSS: Positive and Negative Syndrome Scale.

**Table 2:** Metabolic parameters stratified by nighttime illuminance measured using an actigraph

		**Quartile groups of nighttime illuminance measured using an actigraph**				
**Characteristics**	**Total (n = 222)**	**Q1 n = 55 (<0.5 lux)**	**Q2 n = 56 (0.5 to 1.4 lux)**	**Q3 n = 55 (1.4 to 4.5 lux)**	**Q4 n = 56 (≥ 4.5lux)**	***P trend***
**Anthropometric measurement**						
Obesity, n (%)	123 (55.4%)	19 (34.5%)	34 (60.7%)	31 (56.4%)	39 (69.6%)	<0.01
Height, cm, median (IQR)	162.6 (155.9-170.2)	162.6 (158.3-169.4)	162.5 (157.0-168.9)	161.1 (154.2-167.9)	164.5 (155.0-173.9)	0.84
Body weight, kg, median (IQR)	67.2 (59.6-79.1)	63.0 (54.8-72.7)	67.2 (58.1-78.5)	67.0 (61.2-79.7)	72.8 (61.6-89.6)	<0.01
Abdominal circumference, cm, median (IQR)	94.0 (84.0-102.7)	89.3 (80.0-98.6)	96.7 (85.3-102.5)	94.5 (85.0-104.0)	98.5 (84.0-110.1)	<0.01
Body mass index, kg/m^2^, median (IQR)	25.6 (22.7-29.4)	23.7 (21.5-25.9)	26.0 (23.0-29.8)	26.2 (23.3-29.3)	28.2 (23.5-30.9)	<0.01
**Blood pressure**						
Hypertension, n (%)	95 (42.8%)	16 (29.1%)	24 (42.9%)	22 (40.0%)	33 (58.9%)	<0.01
Systolic blood pressure, mmHg	125.0 (113.0-139.0)	118.0 (110.5-133.5)	126.5 (113.3-137.8)	124.0 (111.0-137.0)	134.0 (120.3-145.3)	<0.01
Diastolic blood pressure, mmHg	82.0 (75.0-92.0)	79.0 (72.0-88.0)	83.0 (76.3-91.0)	81.0 (73.0-90.0)	84.5 (77.0-95.0)	0.02
**Blood sugar**						
Diabetes, n (%)	29 (13.1%)	6 (10.9%)	9 (16.1%)	4 (7.3%)	10 (17.9%)	0.55
Casual plasma glucose, mg/dl	103.0 (92.0-120.0)	98.0 (87.8-111.8)	103.5 (93.0-123.0)	101.5 (92.3-120.0)	105.5 (95.0-134.3)	0.01
HbA1c, %	5.4 (5.2-5.8)	5.3 (5.0-5.5)	5.4 (5.2-5.8)	5.3 (5.1-5.8)	5.5 (5.2-5.9)	<0.01
**Lipids**						
Dyslipidemia, n (%)	108 (48.6%)	23 (41.8%)	24 (42.9%)	24 (43.6%)	37 (66.1%)	0.01
TG, mg/dl	134.0 (88.0-205.0)	105.0 (74.3-179.8)	146.0 (109.0-199.0)	134.0 (92.5-194.0)	169.5 (100.8-295.5)	<0.01
HDL-C, mg/dl	51.0 (42.0-64.5)	61.5 (47.0-77.0)	53.0 (37.8-64.0)	51.0 (43.0-61.0)	45.0 (38.0-57.0)	<0.01
LDL-C, mg/dl	88.3 (72.6-111.0)	83.3 (65.8-100.9)	87.7 (75.3-106.7)	94.9 (73.6-113.2)	95.5 (76.5-119.4)	0.01

Data are expressed as the median (interquartile range) or number (%). CI, confidence interval; HbA1c, Glycosylated hemoglobin; TG, Triglyceride; HDL-C, High-density lipoprotein cholesterol; LDL-C, Low-density lipoprotein cholesterol.

**Figure 2:**
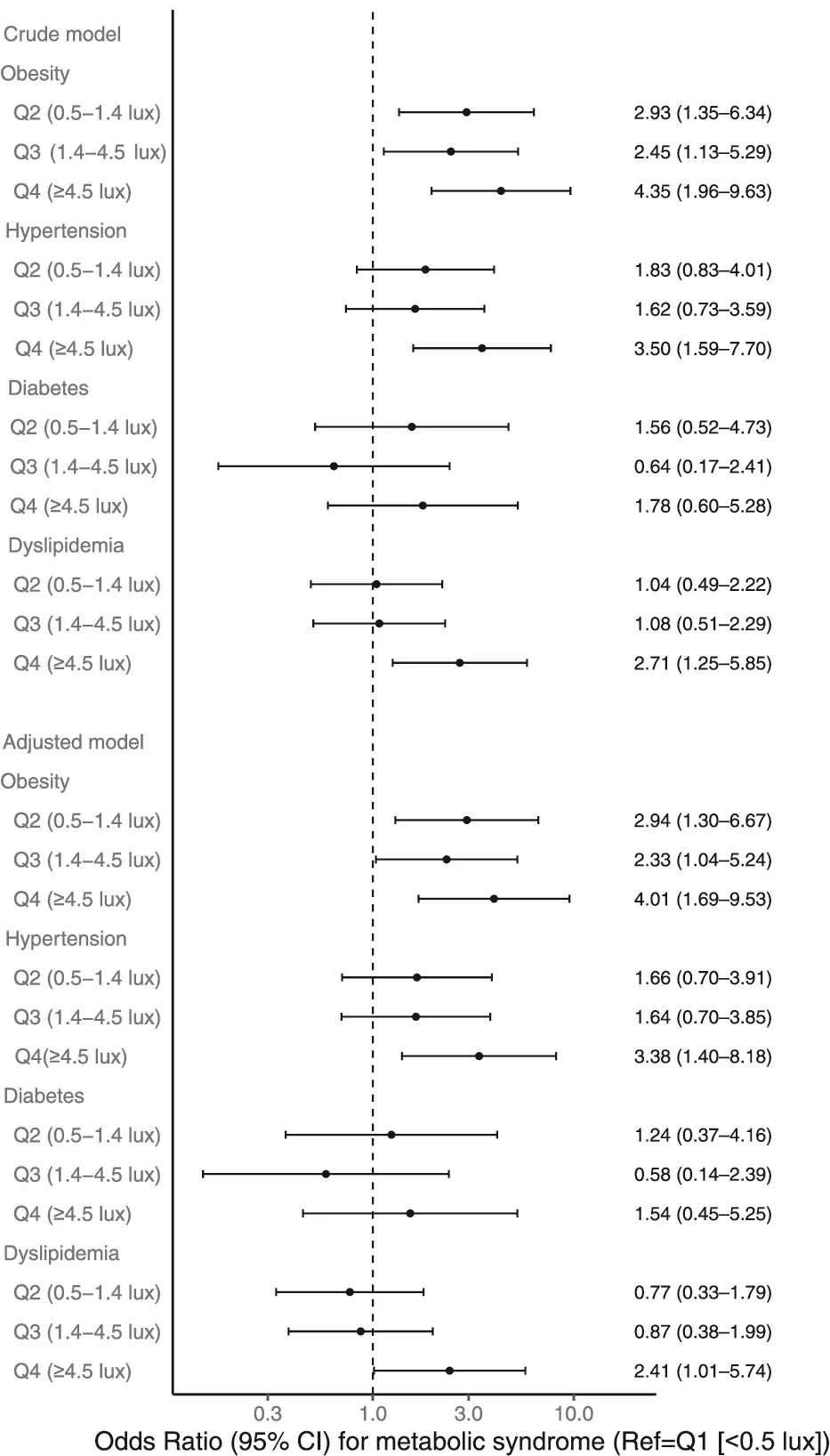
*Logistic regression analysis results for metabolic dysfunction associated with nighttime illuminance quartile measured using an actigraph*. Associations between nighttime illuminance quartile and metabolic dysfunction, including obesity, hypertension, diabetes, and dyslipidemia are illustrated. The model was adjusted for age, gender, smoking status, total sleep time, daytime physical activity, rising time, antipsychotic dose, and antidepressant use. Dots and error bars indicate odds ratios and 95 percent confidence intervals for each nighttime illuminance quartile in relation to the lowest quartile (Q1).

Logistic regression analysis revealed that the highest quartile nighttime illuminance group (Q4) demonstrated significantly higher ORs for obesity (OR, 4.35; 95% confidence interval [CI], 1.96–9.63), hypertension (OR, 3.50; 95% CI, 1.59–7.70), and dyslipidemia (OR, 2.71; 95% CI, 1.25–5.85) than the lowest quartile nighttime illuminance group measured using actigraphy (Q1) (Figure 2, crude model). After adjusting for potential confounding factors, including age, gender, current smoking status, total sleep time, daytime physical activity, rising time, antipsychotic dose, and antidepressant use, the multivariable analysis revealed consistent results (Figure 2, adjusted model). Furthermore, Analysis of covariance revealed that the highest quartile nighttime illuminance group demonstrated significantly higher body weight (+10.3 kg), abdominal circumference (+8.5 cm), BMI (+3.4 kg/m^2^), SBP (+10.1 mmHg), DBP (+6.0 mmHg), HbA1c (+0.37 percent), TG (+77.0 mg/dl), and LDL-C (+13.4 mg/dl) and significantly lower HDL-C (−8.5 mg/dl) than the lowest quartile nighttime illuminance group (Table 3, adjusted model). After FDR correction, these associations remained significant (Table S3).

**Table 3:** Analysis of covariance for metabolic parameters associated with nighttime illuminance quartiles measured using an actigraph

**Metabolic parameters**	**Q1 n = 55, <0.5 lux**	**Q2 (n = 56, 0.5 to 1.4 lux) vs. Q1, difference (95% CI)**	***p***	**Q3 (n = 55, 1.4 to 4.5 lux) vs. Q1, difference (95% CI)**	***p***	**Q4 (n = 56, ≥ 4.5lux) vs. Q1, difference (95% CI)**	***p***
**Crude model**							
Body weight, kg	Ref	5.01 (-1.23 to 11.24)	.12	7.10 (0.84 to 13.36)	.03	11.84 (5.60 to 18.07)	<.01
Abdominal circumference, cm	Ref	6.16 (0.78 to 11.54)	.03	7.69 (2.28 to 13.09)	<.01	10.62 (5.24 to 16.00)	<.01
Body mass index, kg/m2	Ref	2.02 (-0.06 to 4.10)	.06	3.65 (1.56 to 5.74)	<.01	4.06 (1.97 to 6.14)	<.01
Systolic blood pressure, mmHg	Ref	5.33 (-2.08 to 12.75)	.16	3.94 (-3.51 to 11.39)	.30	10.56 (3.15 to 17.98)	<.01
Diastolic blood pressure, mmHg	Ref	3.35 (-1.27 to 7.97)	.15	2.01 (-2.63 to 6.65)	.39	6.34 (1.71 to 10.96)	<.01
Casual plasma glucose, mg/dl	Ref	11.72 (-0.39 to 23.84)	.06	10.87 (-1.25 to 22.99)	.08	17.44 (5.33 to 29.56)	<.01
HbA1c, %	Ref	0.24 (-0.03 to 0.52)	.08	0.11 (-0.16 to 0.38)	.44	0.42 (0.15 to 0.69)	<.01
TG, mg/dl	Ref	27.15 (-36.39 to 90.69)	.40	20.42 (-43.42 to 84.26)	.53	86.13 (22.59 to 149.67)	<.01
HDL-C, mg/dl	Ref	-9.13 (-15.79 to -2.47)	.01	-9.26 (-16.02 to -2.50)	<.01	-12.74 (-19.40 to -6.08)	<.01
LDL-C, mg/dl	Ref	5.68 (-4.76 to 16.12)	.29	9.44 (-1.05 to 19.93)	.08	10.03 (-0.41 to 20.47)	.06
**Adjusted model**							
Body weight, kg	Ref	4.48 (-1.51 to 10.47)	.14	6.91 (0.93 to 12.88)	.02	10.37 (4.19 to 16.55)	<.01
Abdominal circumference, cm	Ref	4.55 (-0.92 to 10.03)	.10	6.68 (1.21 to 12.14)	.02	8.53 (2.87 to 14.18)	<.01
Body mass index, kg/m^2^	Ref	1.74 (-0.41 to 3.89)	.11	3.17 (1.02 to 5.32)	<.01	3.41 (1.19 to 5.63)	<.01
Systolic blood pressure, mmHg	Ref	5.35 (-2.04 to 12.73)	.16	4.90 (-2.46 to 12.26)	.19	10.14 (2.53 to 17.76)	<.01
Diastolic blood pressure, mmHg	Ref	2.87 (-1.93 to 7.67)	.24	1.96 (-2.83 to 6.74)	.42	6.08 (1.13 to 11.04)	.02
Casual plasma glucose, mg/dl	Ref	6.53 (-5.62 to 18.68)	.29	8.80 (-3.28 to 20.88)	.15	11.22 (-1.27 to 23.70)	.08
HbA1c, %	Ref	0.20 (-0.08 to 0.48)	.15	0.05 (-0.22 to 0.33)	.71	0.37 (0.08 to 0.65)	.01
TG, mg/dl	Ref	11.89 (-54.48 to 78.25)	.72	7.88 (-58.38 to 74.13)	.82	77.03 (8.85 to 145.21)	.03
HDL-C, mg/dl	Ref	-4.74 (-11.32 to 1.84)	.16	-5.96 (-12.59 to 0.66)	.08	-8.50 (-15.27 to -1.73)	.01
LDL-C, mg/dl	Ref	7.22 (-3.61 to 18.05)	.19	10.81 (0.00 to 21.62)	.05	13.43 (2.31 to 24.56)	.02

Adjusted model: Adjusting for age, gender, smoke status, total sleep time, daytime physical activity, rising time, antipsychotic dose, and use of antidepressants. CI, confidence interval; HbA1c, Glycosylated hemoglobin; TG, Triglyceride; HDL-C, High-density lipoprotein cholesterol; LDL-C, Low-density lipoprotein cholesterol.

The highest quartile nighttime illuminance group, measured using a bedside photometer, had significantly higher ORs for obesity, hypertension, and dyslipidemia than the lowest quartile nighttime illuminance group (Figure S1, crude model). After adjusting for potential confounding factors, nighttime illuminance was significantly associated with OR for hypertension (Figure S1, adjusted model). Analysis of covariance revealed that the highest quartile nighttime illuminance group showed significantly higher body weight, abdominal circumference, BMI, SBP, DBP, and TG and significantly lower HDL-C than the lowest quartile nighttime illuminance group (Table S1, crude model). After adjusting for potential confounding factors, a significant association was observed between nighttime illuminance and body weight, abdominal circumference, BMI, SBP, and TG (Table S4, adjusted model). However, these associations were no longer significant after FDR correction (Table S5).

The possibility of reverse causation was further evaluated using additional multivariable analyses. After adjustment for confounding factors associated with nighttime light exposure, including age, gender, current smoking status, duration of illness, total sleep time, physical activity, and antipsychotic dose, the highest quartile of nighttime illuminance measured using actigraphy (Q4) was significantly associated with higher odds of obesity, hypertension, and dyslipidemia compared with the lowest quartile (Q1) (Table S6). Meanwhile, increased nighttime illuminance measured using a bedside photometer was significantly associated with hypertension only (Table S7).

## Discussion

To the best of our knowledge, this is the first study to investigate the association between nighttime light exposure and metabolic dysfunction in patients with schizophrenia. The results showed that increased nighttime light exposure, particularly when measured using actigraphy, was significantly associated with higher ORs for obesity, hypertension, and dyslipidemia, including higher body weight, abdominal circumference, BMI, SBP, DBP, TG, and LDL-C and lower HDL-C in patients with schizophrenia. Although no significant association was observed between nighttime illuminance and OR for diabetes, increased nighttime illuminance was significantly associated with higher HbA1c levels. The multivariable analyses revealed that these associations were independent of several potential confounding factors.

The study results are consistent with those of previous studies, which showed that nighttime illuminance was associated with metabolic dysfunction, including obesity, hypertension, and dyslipidemia, in the general population. Previous studies have reported cross-sectional and longitudinal associations between nighttime illuminance measured by a photometer and higher ORs for obesity, including higher body weight, abdominal circumference, and BMI, and dyslipidemia, including higher TG and LDL-C and lower HDL-C, in older adults [17, 18, 20]. Another study reported cross-sectional associations between nighttime illuminance measured using an actigraph and higher ORs for obesity and hypertension among older adults in the United States [13]. Similar findings have been observed for the association between outdoor artificial light at night measured using Satellite data and hypertension in China [15], and these findings were supported by a recent meta-analysis [14]. Our findings support those of previous studies and provide new evidence of the association between increased nighttime light exposure and metabolic dysfunction, including obesity, hypertension, and dyslipidemia, in patients with schizophrenia.

We hypothesized that the association between metabolic dysfunction and nighttime light exposure would be similar when measured using actigraphy and a bedside photometer. In this study, moderate to high correlations were noted between actigraphy- and photometer-measured 7-day nighttime illuminance. Nevertheless, the associations between metabolic dysfunction and nighttime light exposure were stronger when nighttime light exposure was measured using actigraphy than when measured using a bedside photometer. After FDR correction, associations were significant only for actigraphy-measured nighttime illuminance. Wrist-worn actigraphs accurately assess light exposure even when being out of bed, such as when going to the bathroom. However, they have some artifacts due to the possibility of being covered with a bedsheet or clothing. Conversely, a photometer can accurately assess light exposure in a bedroom. However, it cannot assess light exposure when participants get out of bed during the sleep period and go to other rooms, such as the bathroom. Therefore, we assume that the effect of nighttime light exposure was greater for other room lights, such as bathroom lights, than for bedroom lights.

Our hypothesis that nighttime light exposure would be associated with diabetes could not be supported by our results. Previous studies have shown that nighttime light exposure was associated with diabetes in the general population [13, 14, 16, 19]. Conversely, this study did not find any significant association between nighttime light exposure and diabetes in patients with schizophrenia, which may be due to the small sample size. Most previous studies included >1000 participants. In this study, only 29 participants (13.4 percent) had diabetes. Furthermore, this study showed a significant association between nighttime illuminance measured using the actigraph and HbA1c. Therefore, further studies with a larger sample size are needed to clarify the association between nighttime light exposure and diabetes in patients with schizophrenia.

The findings of this study indicate that maintaining darkness during sleep can be a novel clinical therapeutic option for metabolic dysfunction in patients with schizophrenia. People with schizophrenia have a lower life expectancy than the general population, primarily because of premature cardiovascular disease [36, 37]. This may be attributed to a high incidence of metabolic dysfunction in these patients, which is associated with a series of risk factors for cardiovascular disease, such as central obesity, atherogenic dyslipidemia, hypertension, and impaired insulin and glucose metabolism [2, 3]. Therefore, maintaining darkness during sleep may have a beneficial effect on metabolic dysfunction, thereby increasing life expectancy. Further longitudinal controlled studies are needed to confirm the association between nighttime light exposure and metabolic dysfunction in patients with schizophrenia.

The mechanism underlying the association between nighttime light exposure and metabolic dysfunction in patients with schizophrenia remains unknown. However, circadian rhythm dysfunction and abnormal melatonin secretion may be possible mechanisms underlying this association. Light is the primary synchronizer of the central circadian clock [38]. Prolonged exposure to light, particularly during biological night, can disrupt the circadian rhythm and dysregulate clock-regulated physiological processes [39], thereby leading to metabolic dysfunctions, such as elevated body weight, blood pressure, and dyslipidemia. Furthermore, disturbance of melatonin secretion, the “darkness hormone,” may be another mechanism by which nighttime light exposure can impair cardiometabolic function. Nighttime light exposure suppresses pineal melatonin production and weakens the circadian signal that regulates a host of cellular mechanisms [40, 41]. Melatonin plays important roles in metabolic and circulatory functions with antioxidant, anti-inflammatory, and vasodilatory properties [42, 43]. Lower melatonin levels have been reported to be associated with increased risk of obesity, hypertension, and dyslipidemia [44–47]. Therefore, circadian rhythm disruption and abnormal melatonin secretion may mediate the association between nighttime light exposure and metabolic dysfunction in patients with schizophrenia.

The study’s strengths lie in its objective measurement of metabolic dysfunction and in the use of two methods for light exposure measurement, including a wrist-worn actigraph and a bedside photometer. However, this study had several limitations. First, this was a cross-sectional study. We conducted additional analyses adjusting for potential confounders to address the possibility of reverse causation, and the associations remained significant after adjustment. However, causal relationships between nighttime light exposure and metabolic dysfunction could not be inferred. Therefore, further longitudinal studies are needed to investigate the effects of light exposure on metabolic dysfunction in patients with schizophrenia. Second, this study used non-random sampling because participants were recruited from our hospitals or clinics, which limits the generalizability of the findings to all patients with schizophrenia. Third, participants’ adherence to fasting requirements for blood testing could not be confirmed, which might have affected blood test results, particularly plasma glucose and TG levels. Fourth, this study did not capture potential changes in diet habits because no diet questionnaire was specifically designed for patients with mental illnesses. Future studies should include a diet questionnaire to assess the role of diet. Finally, although this study showed that the association between nighttime light exposure and metabolic dysfunction in individuals with schizophrenia was independent of several potential confounding factors, other potential confounders, such as sleep-related breathing disorders and medical conditions, were not considered, which might have introduced confounding bias and affected the results.

This study showed a significant association between nighttime light exposure measured using an actigraph and a bedside photometer and metabolic dysfunction, including obesity, hypertension, and dyslipidemia, in patients with schizophrenia. Further longitudinal studies are needed to investigate the causal relationships between nighttime light exposure and metabolic dysfunction.

## Supplementary Material

2026-6-24_Supplemental_Data_zsag177

## Data Availability

The data that support the findings of this study are available from the corresponding author upon reasonable request.
