# Supplementary material for: Nighttime light exposure is associated with metabolic dysfunction in schizophrenia: a cross-sectional analysis of the LENS study
Source: Sleep. 2026 Jun 29;49(9):zsag177. doi: 10.1093/sleep/zsag177 (PMC13553288; doi:10.1093/sleep/zsag177)
Supplement: Supplementary file 1 [file 2026-6-24_supplemental_data_zsag177.docx]

**Nighttime light exposure is associated with metabolic dysfunction in schizophrenia: A cross-sectional analysis of the LENS study**

Rina Taniguchi ^a,†^,Yuichi Esaki ^a,b,†,*^, Kenji Obayashi ^c^, Keigo Saeki ^c^, Soji Tsuboi ^a^, Kiyoshi Fujita ^a,d^, Nakao Iwata ^b^, Tsuyoshi Kitajima ^b^

^a^Department of Psychiatry, Okehazama Hospital, Aichi, Japan

^b^Department of Psychiatry, Fujita Health University School of Medicine, Aichi, Japan

^c^Department of Epidemiology, Nara Medical University School of Medicine, Nara, Japan

^d^The Neuroscience Research Center, Aichi, Japan

^†^These authors have contributed equally to this work.

*Corresponding author: Yuichi Esaki, MD, PhD

Department of Psychiatry, Fujita Health University School of Medicine, Toyoake, Aichi 4701192, Japan

Phone: +81-562-93-9250; Fax: +81-562-93-1831

Email: [esakiz@fujita-hu.ac.jp](mailto:esakiz@fujita-hu.ac.jp)

**Supplemental Figure 1. Logistic regression analysis results for metabolic dysfunction associated with nighttime illuminance quartile measured using portable photometer**

Figure presents the associations between nighttime illuminance quartile and metabolic dysfunction, including obesity, hypertension, diabetes, and hyperlipidemia. The model was adjusted for age, gender, smoking status, total sleep time, daytime physical activity, rising time, antipsychotic dose, and antidepressant use. Dots and error bars indicate odds ratios and 95% confidence intervals for each nighttime illuminance quartile in relation to the lowest quartile (Q1).

| **Supplemental Table 1.** Spearman’s rank correlation coefficients between the actigraphy- and photometer-measured nighttime illuminance over 7 consecutive days | | | | | | | |
| --- | --- | --- | --- | --- | --- | --- | --- |
|  | Day 1 | Day 2 | Day 3 | Day 4 | Day 5 | Day 6 | Day 7 |
| Actigraphy vs. photometer, *rs* | 0.68 | 0.55 | 0.67 | 0.70 | 0.69 | 0.67 | 0.68 |

| **Supplemental Table 2.** Concordance matrix of Q1–Q4 classifications between actigraphy and photometer measures of nighttime illuminance | | | | | |
| --- | --- | --- | --- | --- | --- |
|  |  | Actigraphy | | | |
|  |  | Q1, <0.5 lux | Q2, 0.5 to 1.4 lux | Q3, 1.4 to 4.5 lux | Q4 ≥ 4.5 lux |
| Bedside photometer | Q1, <1.0 lux | 34 | 10 | 8 | 2 |
|  | Q2, 1.0 to 4.0 lux | 15 | 29 | 9 | 2 |
|  | Q3, 4.0 to 15.4 lux | 4 | 12 | 23 | 16 |
|  | Q4, ≥ 15.4 lux | 1 | 3 | 15 | 33 |

| **Supplemental Table 3. False discovery rate-adjusted p values for associations between nighttime illuminance quartiles measured using actigraphy and metabolic parameters** | | | | |
| --- | --- | --- | --- | --- |
| **Metabolic parameters** | **Q1 (<0.5 lux)** | **Q2 (0.5 to 1.4 lux) vs. Q1, Adjusted q (FDR)** | **Q3 (1.4 to 4.5 lux) vs. Q1, Adjusted q (FDR)** | **Q4 (≥ 4.5 lux) vs. Q1, Adjusted q (FDR)** |
| **Adjusted model** |  |  |  |  |
| **Anthropometric measurement** |  |  |  |  |
| Body weight, kg | Ref | 0.26 | 0.08 | 0.01 |
| Abdominal circumference, cm | Ref | 0.26 | 0.08 | 0.01 |
| Body mass index, kg/m2 | Ref | 0.26 | 0.04 | 0.01 |
| **Blood pressure** |  |  |  |  |
| SBP, mmHg | Ref | 0.26 | 0.27 | 0.02 |
| DBP, mmHg | Ref | 0.30 | 0.53 | 0.02 |
| **Blood sugar** |  |  |  |  |
| Casual plasma glucose, mg/dl | Ref | 0.32 | 0.25 | 0.08 |
| HbA1c, % | Ref | 0.26 | 0.79 | 0.02 |
| **Lipids** |  |  |  |  |
| TG, mg/dl | Ref | 0.72 | 0.81 | 0.03 |
| HDL-C, mg/dl | Ref | 0.26 | 0.15 | 0.02 |
| LDL-C, mg/dl | Ref | 0.27 | 0.12 | 0.02 |
| Adjusted model: Adjusting for age, gender, smoking status, total sleep time, daytime physical activity, rising time, antipsychotic dose, and antidepressant use. CI, confidence interval. | | | | |

| **Supplemental Table 4. Analysis of covariance for metabolic parameters associated with quartiles of nighttime illuminance measured using a bedside photometer** | | | | | | | |
| --- | --- | --- | --- | --- | --- | --- | --- |
| **Metabolic parameters** | **Q1 n=54, <1.0 lux** | **Q2 (n=55, 1.0 to 4.0 lux) vs. Q1, Difference (95% CI)** | ***P*** | **Q3 (n=55, 4.0 to 15.4 lux) vs. Q1, Difference (95% CI)** | ***P*** | **Q4 (n=52, ≥ 15.4 lux) vs. Q1, Difference (95% CI)** | ***P*** |
| **Crude model** |  |  |  |  |  |  |  |
| Body weight, kg | Ref | 2.36 (-4.06 to 44.77) | 0.47 | 1.96 (-4.45 to 8.38) | 0.55 | 8.92 (2.42 to 15.43) | 0.01 |
| Abdominal circumference, cm | Ref | 3.24 (-2.32 to 8.80) | 0.25 | 2.79 (-2.77 to 8.35) | 0.32 | 8.46 (2.82 to 14.09) | <0.01 |
| Body mass index, kg/m2 | Ref | 1.14 (-1.03 to 3.30) | 0.30 | 1.30 (-0.87 to 3.46) | 0.24 | 2.83 (0.63 to 5.02) | 0.01 |
| Systolic blood pressure, mmHg | Ref | 3.93 (-3.57 to 11.43) | 0.30 | 3.58 (-3.92 to 11.09) | 0.35 | 10.13 (2.53 to 17.74) | 0.01 |
| Diastolic blood pressure, mmHg | Ref | 3.64 (-1.04 to 8.32) | 0.13 | 3.73 (-0.95 to 8.42) | 0.12 | 6.03 (1.28 to 10.78) | 0.01 |
| Casual plasma glucose, mg/dl | Ref | 4.23 (-8.39 to 16.84) | 0.51 | 1.94 (-10.56 to 14.45) | 0.76 | 6.40 (-6.40 to 19.21) | 0.33 |
| HbA1c, % | Ref | 0.17 (-0.12 to 0.45) | 0.25 | 0.10 (-0.18 to 0.39) | 0.47 | 0.13 (-0.16 to 0.42) | 0.36 |
| TG, mg/dl | Ref | 28.96 (-36.36 to 94.28) | 0.38 | 32.59 (-31.83 to 97.01) | 0.32 | 90.32 (24.35 to 156.29) | 0.01 |
| HDL-C, mg/dl | Ref | -3.42 (-10.40 to 3.56) | 0.34 | -2.56 (-9.48 to 4.35) | 0.47 | -9.10 (-16.18 to -2.01) | 0.01 |
| LDL-C, mg/dl | Ref | 3.59 (-7.14 to 14.31) | 0.51 | 6.14 (-4.44 to 16.72) | 0.25 | 10.52 (-0.32 to 21.35) | 0.06 |
|  |  |  |  |  |  |  |  |
| **Adjusted model** |  |  |  |  |  |  |  |
| Body weight, kg | Ref | 2.23 (-3.69 to 8.16) | 0.46 | 4.15 (-1.83 to 10.14) | 0.17 | 6.93 (0.72 to 13.13) | 0.03 |
| Abdominal circumference, cm | Ref | 2.89 (-2.55 to 8.33) | 0.30 | 3.55 (-1.94 to 9.04) | 0.20 | 6.17 (0.47 to 11.87) | 0.03 |
| Body mass index, kg/m2 | Ref | 0.98 (-1.15 to 3.11) | 0.37 | 1.42 (-0.73 to 3.57) | 0.19 | 2.30 (0.07 to 4.53) | 0.04 |
| Systolic blood pressure, mmHg | Ref | 4.05 (-3.10 to 11.21) | 0.27 | 4.11 (-3.11 to 11.33) | 0.26 | 7.85 (0.37 to 15.33) | 0.04 |
| Diastolic blood pressure, mmHg | Ref | 3.53 (-1.15 to 8.21) | 0.14 | 4.30 (-0.43 to 9.02) | 0.07 | 4.80 (-0.09 to 9.69) | 0.05 |
| Casual plasma glucose, mg/dl | Ref | 3.68 (-8.29 to 15.64) | 0.55 | 2.68 (-9.28 to 14.65) | 0.66 | 0.04 (-12.49 to 12.57) | 0.99 |
| HbA1c, % | Ref | 0.14 (-0.14 to 0.41) | 0.33 | 0.09 (-0.19 to 0.36) | 0.54 | -0.01 (-0.30 to 0.28) | 0.96 |
| TG, mg/dl | Ref | 28.99 (-36.63 to 94.62) | 0.39 | 31.02 (-34.29 to 96.33) | 0.35 | 85.28 (16.89 to 153.67) | 0.02 |
| HDL-C, mg/dl | Ref | -2.92 (-9.43 to 3.60) | 0.38 | -3.46 (-9.98 to 3.06) | 0.30 | -6.70 (-13.52 to 0.13) | 0.05 |
| LDL-C, mg/dl | Ref | 3.75 (-6.95 to 14.45) | 0.49 | 5.71 (-4.94 to 16.36) | 0.29 | 9.85 (-1.29 to 21.00) | 0.08 |
| Adjusted model: Adjusting for age, gender, smoking status, total sleep time, daytime physical activity, rising time, antipsychotic dose, and antidepressant use . CI, confidence interval; HbA1c, Glycosylated hemoglobin; TG, Triglyceride; HDL-C, High-density lipoprotein cholesterol; LDL-C, Low-density lipoprotein cholesterol. | | | | | | | |

| **Supplemental Table 5. False discovery rate-adjusted p values for associations between nighttime illuminance quartiles measured using bedside photometer and metabolic parameters** | | | | |
| --- | --- | --- | --- | --- |
| **Metabolic parameters** | **Q1 (<1.0 lux)** | **Q2 (1.0 to 4.0 lux) vs. Q1,**  **Adjusted q (FDR)** | **Q3 (4.0 to 15.4 lux) vs. Q1, Adjusted q (FDR)** | **Q4 (≥ 15.4 lux) vs. Q1, Adjusted q (FDR)** |
| **Adjusted model** |  |  |  |  |
| **Anthropometric Measurement** | |  |  |  |
| Body weight, kg | Ref | 0.54 | 0.42 | 0.08 |
| Abdominal circumference, cm | Ref | 0.54 | 0.42 | 0.08 |
| Body mass index, kg/m2 | Ref | 0.54 | 0.42 | 0.08 |
| **Blood pressure** |  |  |  |  |
| SBP, mmHg | Ref | 0.54 | 0.42 | 0.08 |
| DBP, mmHg | Ref | 0.54 | 0.42 | 0.08 |
| **Blood sugar** |  |  |  |  |
| Casual plasma glucose, mg/dl | Ref | 0.55 | 0.66 | 0.99 |
| HbA1c, % | Ref | 0.54 | 0.60 | 0.99 |
| **Lipids** |  |  |  |  |
| TG, mg/dl | Ref | 0.54 | 0.44 | 0.08 |
| HDL-C, mg/dl | Ref | 0.54 | 0.42 | 0.08 |
| LDL-C, mg/dl | Ref | 0.54 | 0.42 | 0.10 |
| Adjusted model: Adjusting for age, gender, smoking status, total sleep time, daytime physical activity, rising time, antipsychotic dose, and antidepressant use . CI, confidence interval. | | | | |

| **Supplemental Table 6. Logistic regression analysis results for metabolic dysfunction associated with nighttime illuminance quartiles measured using an actigraphy** | | | | | | | |
| --- | --- | --- | --- | --- | --- | --- | --- |
| **Metabolic parameters** | **Q1 <0.5 lux** | **Q2  0.5 to 1.4 lux** | ***P*** | **Q3 1.4 to 4.5 lux** | ***P*** | **Q4 ≥ 4.5lux** | ***P*** |
| **Adjusted model** |  |  |  |  |  |  |  |
| Obesity, OR (95%CI) | Ref | 2.94 (1.30 to 6.69) | 0.01 | 2.39 (1.06 to 5.39) | 0.04 | 4.03 (1.71 to 9.53) | <0.01 |
| Hypertension, OR (95%CI) | Ref | 1.53 (0.63 to 3.75) | 0.35 | 1.25 (0.52 to 3.02) | 0.62 | 2.87 (1.17 to 7.06) | 0.02 |
| Diabetes, OR (95%CI) | Ref | 1.20 (0.35 to 4.11) | 0.77 | 0.46 (0.11 to 1.89) | 0.28 | 1.26 (0.38 to 4.20) | 0.71 |
| Dyslipidemia, OR (95%CI) | Ref | 0.79 (0.35 to 1.82) | 0.58 | 0.92 (0.41 to 2.07) | 0.84 | 2.47 (1.05 to 5.77) | 0.04 |
| Adjusted model: Adjusting for age, gender, smoking status, duration of illness, total sleep time, daytime physical activity, and antipsychotic dose. OR: odds ratio, CI: confidence interval. | | | | | | | |

| **Supplemental Table 7. Logistic regression analysis results for metabolic dysfunction associated with nighttime illuminance quartiles measured using a bedside photometer** | | | | | | | |
| --- | --- | --- | --- | --- | --- | --- | --- |
| **Metabolic parameters** | **Q1 <1.0 lux** | **Q2  1.0 to 4.0 lux** | ***P*** | **Q3 4.0 to 15.4 lux** | ***P*** | **Q4 ≥ 15.4lux** | ***P*** |
| **Adjusted model** |  |  |  |  |  |  |  |
| Obesity, OR (95%CI) | Ref | 1.56 (0.72 to 3.40) | 0.26 | 1.77 (0.81 to 3.89) | 0.15 | 1.91 (0.83 to 4.38) | 0.13 |
| Hypertension, OR (95%CI) | Ref | 2.84 (1.17 to 6.89) | 0.02 | 2.55 (1.03 to 6.27) | 0.04 | 3.04 (1.22 to 7.57) | 0.02 |
| Diabetes, OR (95%CI) | Ref | 2.22 (0.66 to 7.53) | 0.20 | 1.73 (0.49 to 6.12) | 0.39 | 0.94 (0.24 to 3.63) | 0.93 |
| Dyslipidemia, OR (95%CI) | Ref | 1.19 (0.54 to 2.65) | 0.66 | 1.95 (0.88 to 4.35) | 0.10 | 2.21 (0.96 to 5.10) | 0.06 |
| Adjusted model: Adjusting for age, gender, smoking status, duration of illness, total sleep time, daytime physical activity, and antipsychotic dose. OR: odds ratio, CI: confidence interval. | | | | | | | |
